# Supplementary material for: Genome-scale metabolic network reconstruction analysis identifies bacterial vaginosis-associated metabolic interactions
Source: Nat Commun. 2025 May 22;16:4768. doi: 10.1038/s41467-025-59965-y (PMC12098912; doi:10.1038/s41467-025-59965-y)
Supplement: Supplementary file 1 — Supplementary Information [file 41467_2025_59965_MOESM1_ESM.pdf]

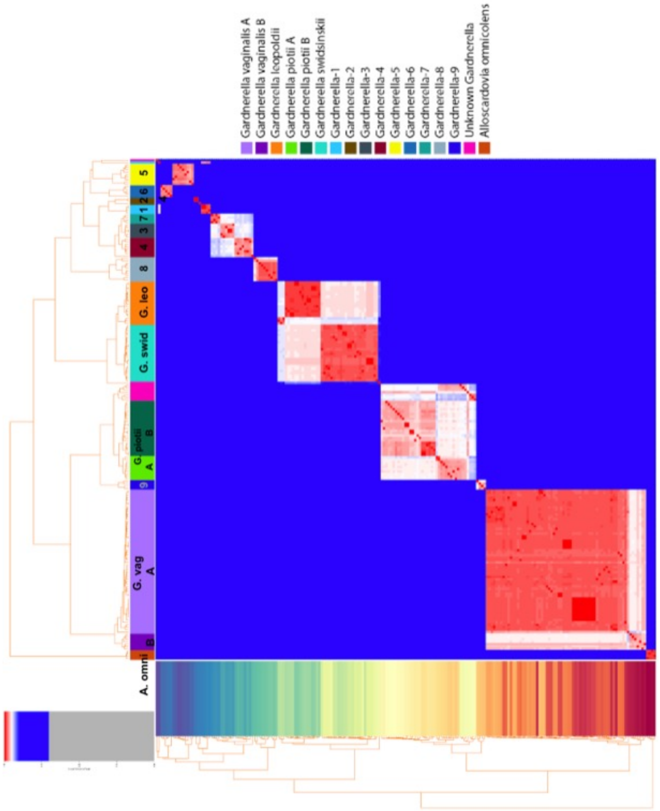

S1 | *Gardnerella* average nucleotide identity (ANI) hierarchically clustered distance matrix

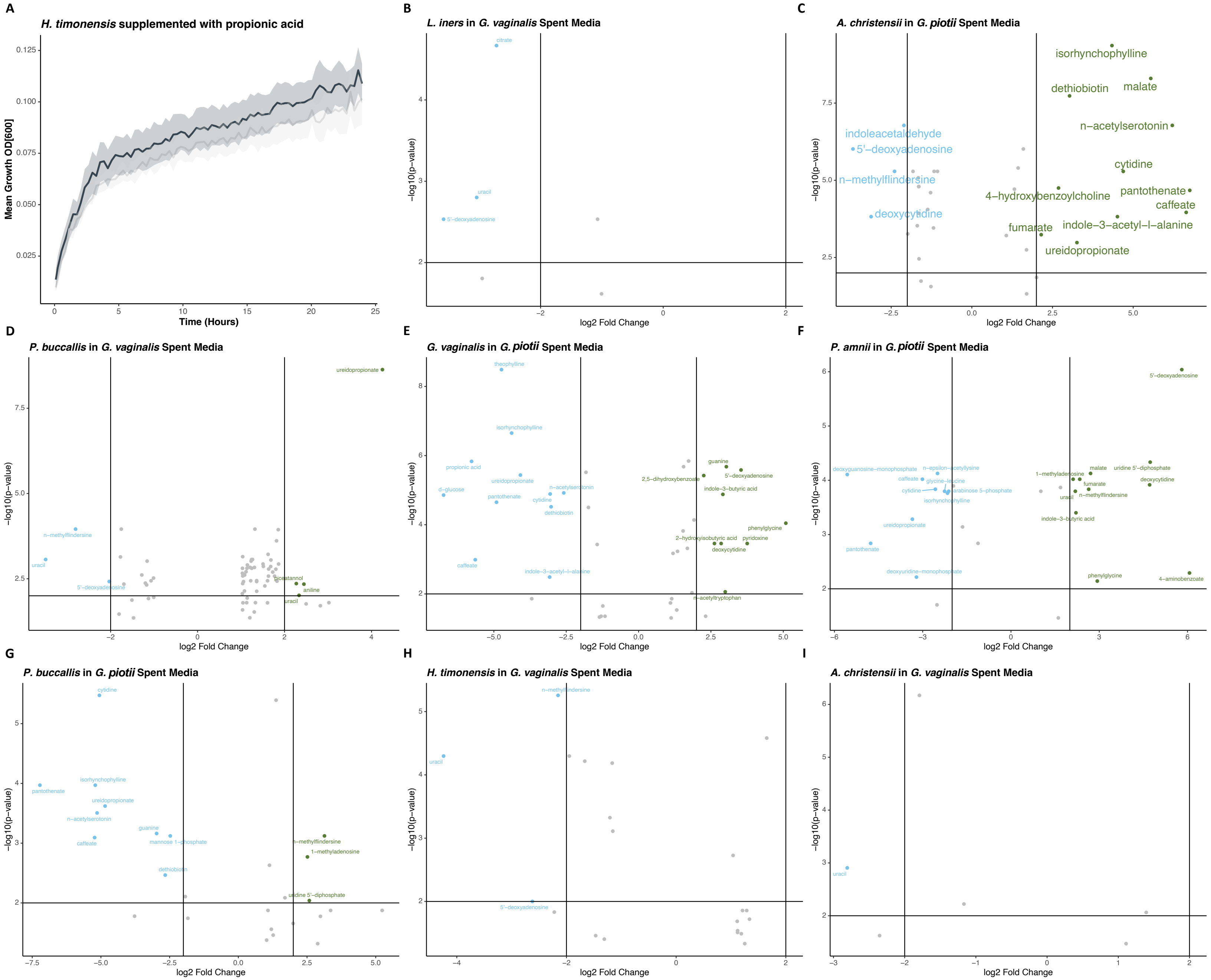

S2 | Supplemented growth curve and differential metabolite plots. (A) Growth curve of *H. timonensis* supplemented with propionic acid. (B-I) Volcano plots of differential metabolites (blue: consumed; green: produced; gray: not significant) from co-occurring species grown in *G. vaginalis spent* media.
