# Supplementary material for: Genome-scale metabolic network reconstruction analysis identifies bacterial vaginosis-associated metabolic interactions
Source: Nat Commun. 2025 May 22;16:4768. doi: 10.1038/s41467-025-59965-y (PMC12098912; doi:10.1038/s41467-025-59965-y)
Supplement: Supplementary file 2 — Description of Additional Supplementary Files [file 41467_2025_59965_MOESM2_ESM.docx]

Description of Additional Supplementary Files

1. **Supplementary Data 1** Database of genomes used for taxonomic classification of MAGs assembled as a result of the present study.
