# Supplementary material for: Genome-scale metabolic network reconstruction analysis identifies bacterial vaginosis-associated metabolic interactions
Source: Nat Commun. 2025 May 22;16:4768. doi: 10.1038/s41467-025-59965-y (PMC12098912; doi:10.1038/s41467-025-59965-y)
Supplement: Supplementary file 4 — Reporting Summary [file 41467_2025_59965_MOESM4_ESM.pdf]

nature portfolio

Corresponding author(s): Jason Papin

Last updated by author(s): Apr 29, 2025

Reporting Summary

Nature Portfolio wishes to improve the reproducibility of the work that we publish. This form provides structure for consistency and transparency in reporting. For further information on Nature Portfolio policies, see our Editorial Policies and the Editorial Policy Checklist.

Statistics

For all statistical analyses, confirm that the following items are present in the figure legend, table legend, main text, or Methods section.

n/a	Confirmed	
☐	☒	The exact sample size (*n*) for each experimental group/condition, given as a discrete number and unit of measurement
☐	☒	A statement on whether measurements were taken from distinct samples or whether the same sample was measured repeatedly
☐	☒	The statistical test(s) used AND whether they are one- or two-sided<br>*Only common tests should be described solely by name; describe more complex techniques in the Methods section.*
☒	☐	A description of all covariates tested
☐	☒	A description of any assumptions or corrections, such as tests of normality and adjustment for multiple comparisons
☐	☒	A full description of the statistical parameters including central tendency (e.g. means) or other basic estimates (e.g. regression coefficient) AND variation (e.g. standard deviation) or associated estimates of uncertainty (e.g. confidence intervals)
☐	☒	For null hypothesis testing, the test statistic (e.g. *F*, *t*, *r*) with confidence intervals, effect sizes, degrees of freedom and *P* value noted<br>*Give P values as exact values whenever suitable.*
☒	☐	For Bayesian analysis, information on the choice of priors and Markov chain Monte Carlo settings
☒	☐	For hierarchical and complex designs, identification of the appropriate level for tests and full reporting of outcomes
☒	☐	Estimates of effect sizes (e.g. Cohen's *d*, Pearson's *r*), indicating how they were calculated

Our web collection on statistics for biologists contains articles on many of the points above.

Software and code

Policy information about availability of computer code

Data collection	Sample collection, processing, shotgun metagenomic sequencing (NovaSeq, Illumina), and bioinformatics analysis was completed as described in the manuscript.
Data analysis	pyani v0.2.12, TAPE v0.3.0, iTOL v6.7.1, BV-BRC 3.28.21 database, Reconstructor v1.1.0, MEMOTE 0.13.0., p.adjust from the R stats package v 3.6.2. Code generated during this study is available in a public Github repository: https://github.com/lrd3uu/bacterialvaginosis_interactions. All metabolic network models generated during this study are available in a public Github repository: https://github.com/emmamglass/Gardnerella-Interactions. All code is also availalable at a stable zenodo doi: https://doi.org/10.5281/ZENODO.15231789.

For manuscripts utilizing custom algorithms or software that are central to the research but not yet described in published literature, software must be made available to editors and reviewers. We strongly encourage code deposition in a community repository (e.g. GitHub). See the Nature Portfolio guidelines for submitting code & software for further information.

Data

Policy information about availability of data

All manuscripts must include a data availability statement. This statement should provide the following information, where applicable:

- Accession codes, unique identifiers, or web links for publicly available datasets
- A description of any restrictions on data availability
- For clinical datasets or third party data, please ensure that the statement adheres to our policy

The raw metabolomics files provided to us by the UVA Metabolomics core were uploaded to the Metabolights repository under the following identifier:

REQ20250415209971. Stable dropbox links to all raw metabolomics files are also available in the github repository in the readme file under the metabolomics heading (https://github.com/lrd3uu/bacterialvaginosis_interactions). The host-depleted raw metagenomic sequencing data (fastq files) are available on the sequence read archive (SRA) under the following BioProject accession number: PRJNA1219227 (https://www.ncbi.nlm.nih.gov/bioproject/?term=(PRJNA1219227)%20AND%20bioproject_sra[filter]%20NOT%20bioproject_gap[filter]). All MAGS used in this study were previously made publicly available and can be accessed via BioProject accession numbers listed in Supplementary Data File S3.

Research involving human participants, their data, or biological material

Policy information about studies with human participants or human data. See also policy information about sex, gender (identity/presentation), and sexual orientation and race, ethnicity and racism.

Reporting on sex and gender	Our study included metagenomic samples collected from people with vaginas, we did not specifically probe potential compositional differences in cis versus trans women in our manuscript.
Reporting on race, ethnicity, or other socially relevant groupings	We did not specifically inspect difference in vaginal composition based on race.
Population characteristics	Due to the nature of the study, samples were exclusively from people with vaginas. Outside of this population, remaining characteristics were the result of recruitment by their physician and desire to participate in the online platform (self-recruitment).
Recruitment	Participants were self-recruited through Evvy's online platform ,which does require a monetary purchase in order to submit one's vaginal sample and have it analyzed. This monetary component could self select for more affluent population.
Ethics oversight	Viome IRB# 20220118.evvy

Note that full information on the approval of the study protocol must also be provided in the manuscript.

Field-specific reporting

Please select the one below that is the best fit for your research. If you are not sure, read the appropriate sections before making your selection.

☒ Life sciences ☐ Behavioural & social sciences ☐ Ecological, evolutionary & environmental sciences

For a reference copy of the document with all sections, see nature.com/documents/nr-reporting-summary-flat.pdf

Life sciences study design

All studies must disclose on these points even when the disclosure is negative.

Sample size	For mass spectrometry analysis we discussed with the metabolomics specialist the field standard for biological and technical replicates, resulting in us submitting 5 biological replicates. Additionally, for growth data we generated 7 biological replicates per condition. No sample size calculation was performed.
Data exclusions	No data was excluded from this analysis.
Replication	We included biological replicates in all of our growth and metabolomic analyses to confirm results hold true across biological replicates. This ensures reproducibility of the study if others attempted to replicate it in the future.
Randomization	Allocation to each growth condition was random by nature of bacterial variation within liquid culture.
Blinding	Blinding was not possible due to personnel constrains and making it unfeasible to have separate persons conduct the experiment and analyze the data.

Reporting for specific materials, systems and methods

We require information from authors about some types of materials, experimental systems and methods used in many studies. Here, indicate whether each material, system or method listed is relevant to your study. If you are not sure if a list item applies to your research, read the appropriate section before selecting a response.

Materials & experimental systems

n/a	Involved in the study
☒	☐ Antibodies
☒	☐ Eukaryotic cell lines
☒	☐ Palaeontology and archaeology
☒	☐ Animals and other organisms
☒	☐ Clinical data
☒	☐ Dual use research of concern
☒	☐ Plants

Methods

n/a	Involved in the study
☒	☐ ChIP-seq
☒	☐ Flow cytometry
☒	☐ MRI-based neuroimaging

Plants

Seed stocks

Report on the source of all seed stocks or other plant material used. If applicable, state the seed stock centre and catalogue number. If plant specimens were collected from the field, describe the collection location, date and sampling procedures.

Novel plant genotypes

Describe the methods by which all novel plant genotypes were produced. This includes those generated by transgenic approaches, gene editing, chemical/radiation-based mutagenesis and hybridization. For transgenic lines, describe the transformation method, the number of independent lines analyzed and the generation upon which experiments were performed. For gene-edited lines, describe the editor used, the endogenous sequence targeted for editing, the targeting guide RNA sequence (if applicable) and how the editor was applied.

Authentication

Describe any authentication procedures for each seed stock used or novel genotype generated. Describe any experiments used to assess the effect of a mutation and, where applicable, how potential secondary effects (e.g. second site T-DNA insertions, mosiacism, off-target gene editing) were examined.
